# HIV prevention by vaccine

**DOI:** 10.1186/1742-4690-9-S1-I5

**Published:** 2012-05-25

**Authors:** Robert Gallo

**Affiliations:** 1 Institute of Human Virology, Baltimore, USA

## 

Three major HIV vaccine efficacy clinical trials have now been completed. Two failed, one of the two even increased infection and the third was modestly successful. First: VaxGen, used conventional gp120 protein and like in monkey trials it failed likely due to type specific Abs and perhaps inadequate Ab titans; the second, by the Vaccine Research Center (VRC) and Merck based solely on CMI and predictably failed. Less predictably it actually increased the numbers infected. This was likely due to use as a vector of an adenovirus strain already exposed to a sizeable percentage of people from earlier infections, thereby leading to increased T-cell activation which is accompanied by an increase in CCR5 co-receptor for HIV and consequently to increased susceptibility to HIV infection. The third large efficacy trial involved a novel gp120 delivered by the canary pox virus known as ALVAC made by Sanofi as well as some other HIV genes and boosted by a gp120 containing a herpes virus small sequence known as Gd. This trial was run by Colonel Dr. Nelson Michael and his co-workers in the U.S. Army AIDS research group in collaboration with colleagues in Thailand and resulted in modest success associated with Abs (not CMI) which binds V2 of gp120 and without detectable neutralizing Ab activity. Of great interest to us was the short duration of the Abs resulting in far greater success in the first half year than in the remaining part of the study. This is typical for Abs to gp120, and it is precisely what we have found in our primate challenge experiments with our candidate vaccine, a complex of gp120 with binding region of CD4 which we (A. DeVico, G. Lewis, T. Fouts, and Y. Guan) call the full length single chain (FLSC).

I will summarize our rationale for the FLSC, our updated primate results, and our plans for clinical trials in collaboration with Sanofi, N. Michael and his group, and the Gates Foundation.

